# The Psychology of Resistance to Change: The Antidotal Effect of Organizational Justice, Support and Leader-Member Exchange

**DOI:** 10.3389/fpsyg.2021.678952

**Published:** 2021-08-02

**Authors:** Nabeel Rehman, Asif Mahmood, Muhammad Ibtasam, Shah Ali Murtaza, Naveed Iqbal, Edina Molnár

**Affiliations:** ^1^School of Accountancy & Finance, The University of Lahore, Lahore, Pakistan; ^2^Department of Business Studies, Namal Institute, Mianwali, Pakistan; ^3^Institute of Business & Management, University of Engineering and Technology, Lahore, Pakistan; ^4^Institute of Management and Organizational Sciences, University of Debrecen, Debrecen, Hungary; ^5^Department of Business Administration, University of the Punjab, Lahore, Pakistan

**Keywords:** organizational justice, distributive justice, procedural justice, interactional justice, perceived organizational support, leader-member exchange, readiness for change, resistance to change

## Abstract

In today’s business environment, the survival and sustenance of any organization depend upon its ability to introduce a successful change. However, in implementing a change, one of the biggest problems an organization faces is resistance from its employees. The current paper addresses this problem by examining the role of organizational justice dimensions in coping with the resistance to change through the intervening role of perceived organizational support (POS), leader-member exchange (LMX), and readiness for change (RFC) in a sequential framework. Data of 372 employees have been collected from the banking industry of Pakistan. The results obtained through the Partial Least Squares- Structural Equation Modeling (PLS-SEM) approach using SmartPLS suggest that distributive justice, procedural justice, and interactional justice play a critical role in lowering the resistance to change through POS, LMX, and RFC, contributing significantly to the theory and practice. Furthermore, this study also discusses recommendations for future research and limitations associated with this research work.

## Introduction

Since today’s business environment frequently confronts changing market trends, globalization, and technological advancements, firms need to continuously revisit their processes, strategies, and culture (Cummings and Worley, 2009; Petrou et al., 2018). Over time, a review of change management has acknowledged the importance of organizational change (Burnes and Jackson, 2011). Consistency in introducing change has arguably become a key to survival (McKinsey Company, 2008; Burnes, 2009). Therefore, organizations are under constant pressure to initiate and execute organizational change (Shah, 2011). In developing countries like Pakistan, the financial services industry also faces competitive challenges for their survival and sustenance. Several banks have gone through enormous changes like mergers and acquisitions, adopting new technology, reforms in business operations, and changes in human resource policies (Osei-Bonsu, 2014). Hence, the financial services institutions must consider introducing change from time to time to stay in business, meet market standards, and maintain a competitive edge.

However, change processes are pretty challenging, and most organizations struggle to execute change strategies (Burnes, 2009). The literature suggests that more than two-thirds of change implementation efforts fail (Beer and Nohria, 2000; Meaney and Pung, 2008). One of the most critical failures to change is employees’ attitudes toward change (Ahmad and Cheng, 2018). Unaware of the potential benefits associated with the organizational change, employees often develop a sense of fear, and perceive the introduction of change as an unfair act (Ford and Ford, 2010). Therefore, they develop negative attitudes and exhibit adverse reactions toward change—a phenomenon known as resistance to change (RTC) (Folger and Konovsky, 1989). Thus, shaping the employees’ resistive attitudes is considered vital for success in implementing change. A recent study by Banguntopo (2018) has drawn our attention to the factors influencing RTC and suggested that the employees’ readiness for change (RFC) greatly influences RTC by transforming their attitudes in favor of the change. Some early research has established that the employees’ beliefs, attitudes, and intentions toward organizational change determine their state of RFC (Armenakis et al., 1993), furthermore, the RFC depends upon the employees’ behaviors and emotions toward change (Oreg, 2003).

Based on the above discussion, it can be argued that the success of change execution effort largely depends on shaping employee attitudes toward change, i.e., coping with RTC by making the employees ready for change. Currently, considerable research work acknowledges the importance of change management in employee response, and the factors influencing those responses. Some recent studies have highlighted the importance of organizational justice practices in shaping employee response toward change (Soenen and Melkonian, 2017). It is believed that the perception of organizational justice greatly influences the beliefs, attitudes, intentions, behaviors, and emotions of employees. For instance, if employees perceive that the management does justice in outcomes, rewards distribution (distributive justice), is honest in its procedures and policies (procedural justice) regarding outcomes and rewards, and is fair in the communication process regarding distributions and procedures (interactional justice), they are more likely to show the RFC. In addition, if employees perceive that their organization is supportive, they reciprocate their support in response—a phenomenon known as perceived organizational support (POS), thereby developing a positive attitude in the context of organizational change (Ciliana and Mansoer, 2008). Accordingly, if an employee perceives that his boss treats him well, he will likely be well-motivated, committed, and willing to accept whatever his organization entrusts him—known as Leader-Member Exchange (LMX). Such social exchange relationships can derive change implementation process toward success (Niehoff et al., 2001).

Thus, employee’s justice perception, POS and LMX hold sheer importance in the context of RFC and RTC, however, a review of the literature suggests that there is a dearth of knowledge in this area. First, the previous studies have focused on general change antecedents such as employee commitment, employee beliefs, and job satisfaction (Madsen et al., 2005; Bernerth et al., 2007). Second, most researchers have considered organizational justice as a whole, but less attention was given to the dimensions of justice in the context of organizational change (Arnéguy et al., 2018). Third, the underlying mechanism in a justice-change relationship involving social exchange links is yet to be explored (Nova and Hadiyan, 2017). In fact, very limited studies have examined the justice-change relationship comprehensively (Shah, 2011; Arnéguy et al., 2018; Mangundjaya, 2020). Moreover, no research has explored the intervening roles of POS, LMX, and RFC between the relationship of justice dimensions and RTC in sequential order.

In this backdrop, the present study contributes by developing the underlying mechanisms that examine the impact of justice dimensions (distributive justice, procedural justice, and interactional justice) on resistance to change (RTC) by empirically analyzing the intervening role of POS, LMX, and RFC in sequence. Secondly, this study contributes to the literature by discussing the results from a theoretical as well as a practical viewpoint to broaden the knowledge base of management and future researchers.

## Background and Hypotheses Development

### Resistance to Change: Why Is It of Concern?

Studies have shown that the success of organizational change primarily relies on the attitude and response of their employees toward change (Ahmad and Cheng, 2018). As a matter of fact, appropriate transformation in employees’ behavior toward change determines its long-term success. As early response and intention toward change are crucial (Bayiz Ahmad et al., 2020), a large body of research supports the role of employees’ positive attitudes in the success of change (Herold et al., 2008). In contrast, employees’ negative attitudes and responses may prove harmful. The phenomenon of resistance to change (RTC) reflects the negative attitudes and behaviors expressed by the employees during times of organizational change. During the change execution process, the biggest challenge faced by the organizations is how to manage that change, especially to cope with the resistance posed by the employees. The employees either try to slow down the change process or terminate the change effort entirely (Hughes, 2006). Hence, resistance is a leading obstacle in the way of an organization’s efforts for improvement, survival, or adoption of new processes and technology. But most of the time, management does not consider employees’ perception about stress or uncertainty associated with the change process, which becomes a major cause of resistance, and may lead the change implementation effort to failure (Ahmad and Cheng, 2018). Hence, to make change process a success, the management must not see resistance as a mere obstacle but an opportunity to learn and subsequently reduce it (Strebel, 1996).

Similarly, it has been found that despite circumstances push for a change, the employees are likely to show resistance by sticking to the notion that they do not need the change (Robins et al., 2011). Robbins and Galperin (2010) state that resisting organizational change is in the nature of employees because they often find it uncomfortable to leave their comfort zone. Employees generally get stressed out due to the fear of the unknown. Yue (2008) argued that the greatest challenge that an organization faces during the change process is to deal with the resistive reaction of employees. For many years, it has been believed that the resistance to change is a counterproductive element that reflects employees’ individual and collective negative responses (Collinson, 1994; King and Anderson, 1995; Waddell and Sohal, 1998; Trader-Leigh, 2002). Since employee resistance is a factor that significantly contributes to the failure of a change (Sirkin et al., 2005), serious research efforts have been undertaken to identify predictors of the resistance, individual and collective perceptions about change, their influence on the resistance, benefits, and threats associated with change (Erwin and Garman, 2010; Colquitt et al., 2013).

The positive perception of justice is among the coping mechanisms of resistance, as it has been argued that the distribution of resources, processes, and procedures influence the employees’ attitude and behavior in the context of change (Ford et al., 2008). In this regard, our study extends the literature by highlighting the role of organizational justice in coping with employee resistance through the lens of social exchange relationships, i.e., POS and LMX. Here, it is argued that if management observes fairness and justice in distribution procedures and processes, a message of fairness would be delivered throughout the organization, which will shape the employees’ perception in enforcing openness for the change. The following section hypothesizes the relationships between dimensions of organizational justice, POS, LMX, employee RFC, and resistance to change.

### Distributive Justice and Perceived Organizational Support

Perceived Organizational Support (POS) refers to the perception of employees about how their organization appraises their efforts, and takes care of their welfare, social needs, and career development. Generally, POS is about how an organization extends its support to its employees, and this organization-oriented support enhances their commitment level in return (Baran et al., 2012). POS draws its roots from social exchange theory which suggests that it is a mutual relationship between an organization and its employees, for instance, if an employee perceives that his organization supports him, he will formulate a strong connection with his organization, and participate in extra-role activities to realize the organizational goals. POS can be enhanced through organizational justice, growth opportunities, and support from supervisors and coworkers (Fu and Lihua, 2012; Cheung, 2013; Jacobs et al., 2014).

Previously, there has been a rise in studies focusing on the dimensions of organizational justice from a social exchange perspective. The scholars have suggested its impact in determining the quality of social exchange relationships (Colquitt et al., 2013). Among dimensions of organizational justice, distributive justice derives its roots from equity theory (Adams, 1965), and refers to an employee’s perception of the distribution of organizational rewards and outcomes (Niehoff and Moorman, 1993). The employees who believe that their employer does justice in the distribution of outcomes are motivated, committed, and loyal toward their organization. On the contrary, if employees perceive that their employer distributes injustice, they are likely to change their attitude, lower their morale, and may not participate in job activities as desired (Greenberg, 1993). In a study, Fasolo (1995) supported this argument, and another study explicitly demonstrated that distributive justice is related to POS (Shore and Shore, 1995; Kurtessis et al., 2017). The extent to which an organization takes care of its employees determines employee perception about the organizational support (Loi et al., 2006; Fu and Lihua, 2012). Therefore, it is argued that the employees who perceive their organization has been fair in distributing pay-offs are more likely to contribute toward their organization effectively. The employees with the perception of fair distributive justice show more commitment to their organization, and support it in achieving strategic goals. Based on these arguments, it is hypothesized that:

H_1a_: Distributive Justice positively affects Perceived Organizational Support.

### Distributive Justice and Leader-Member Exchange

Leader-member exchange (LMX) refers to the “exchange outcomes” realized from relationships between an employee and manager, follower and leader, or worker and supervisor (Liden et al., 1993; Scandura, 1999). Here, the word “exchange” indicates that this is a two-way relationship with mutual outcomes. The quality of the relationship between a manager and his employee, and the length of the period of such relationship determine the interpersonal understanding of both parties. If the quality of such a relationship is high, there will be more trust, respect, mutual understanding, and information exchange between the parties. While on the other hand, a low-quality relationship results in a decreased trust level, formality in employee-manager relations, and one-sided influence and manipulation (Bauer and Green, 1996).

While going through the literature, we find that there has been a focus on studies about relationships between dimensions of organizational justice and LMX (Scandura, 1999; Brown et al., 2005). Drawing upon the level of organizational justice, leaders may develop high-quality relationships with some employees, and low-quality relationships with other employees within the organization. The employees who receive better outcomes, rewards and social benefits from their leaders may develop a high-quality LMX relationship (Lee et al., 2010). Therefore, it is argued that distributive justice affects the quality of the LMX relationship: if there is a positive perception of distributive justice among the employees, there will be a strong LMX relationship. Here, it is proposed that:

H_1b_: Distributive Justice Positively affects Leader-Member Exchange.

### Procedural Justice and Perceived Organizational Support

An organization provides benefits or outcomes to its employees such as career counseling, promotions, training and other social benefits to win their loyalty. But unfortunately, situations may arise that even all the provided perks may not be enough to induce the desired impact on the behavior of the employees. This may be the case when an organization does not pay attention to the effectiveness of procedures adopted for the distribution. Hence, procedural justice holds equal importance as distributive justice (DeConinck, 2010). Therefore, it is contended that procedural justice has an impact on POS. Several studies have supported the argument (Loi et al., 2006; Stinglhamber et al., 2006; Peelle, 2007; Fu and Lihua, 2012). Thus, if an organization is fair in procedures and policies adopted for the distribution of outcomes, it will create a positive perception among its employees (Loi et al., 2006). Therefore, drawing upon the literature, it is proposed that as fair distributive procedures preserve the rights of employees in terms of organizational justice, it tends to influence employees’ POS positively. Here, it is hypothesized that:

H_2a_: Procedural Justice positively affects the Perceived Organizational Support.

### Procedural Justice and Leader-Member Exchange

As discussed earlier, an employee with a positive perception of distributive justice tends to form a high-quality LMX relationship. Similarly, his perception of procedures adopted by the organization also matters in determining the quality of LMX. If an employee perceives that the distributive procedures adopted by the organization are justified, he is likely to form a perception that his organization is fair to him. It would help build trust and confidence in his management, ensuing a high-quality LMX. Lee et al. (2010) argued that LMX is related to the dimensions of justice: procedural justice and distributive justice. In some studies, LMX has also been observed to contribute as a moderator in the relationship between procedural justice and distributive justice with specific organizational outcomes (Piccolo et al., 2008). Therefore, deriving from the literature, it is reasoned that procedural justice positively affects the quality of the supervisor-subordinate relationship. Hence, we propose that:

H_2b_: Procedural Justice positively affects the Leader-Member Exchange.

### Interactional Justice and Perceived Organizational Support

Interactional justice is the third dimension of organizational justice that augments the earlier discussed dimensions of justice. It reflects how an organization treats, interacts, and communicates with its employees during the execution process of procedures and distributions (Bies and Moag, 1986). According to organizational support theory, when employees receive recognition for their contributions, they become more loyal to their organization. So, interactional justice imparts a sense of being influential among the employees, which increases their trust in the management and supervisors. Subsequently, it will arguably enhance the perception of organizational support. Organizations with a strong focus on interactional justice will have improved POS relationships than those without it. Despite its importance, the literature indicates that interactional justice has been mostly ignored in the past (Cheung and Law, 2008; DeConinck, 2010). However, in a recent meta-analysis, interactional justice has been found to be positively related to POS (Kurtessis et al., 2017). Hence, based on the literature, it is postulated that employees will find themselves well aware and well communicated with their organization, and show their support for it if interactional justice persists. Thus, it is hypothesized that:

H_3a_: Interactional Justice positively affects the Perceived Organizational Support.

### Interactional Justice and Leader-Member Exchange

The researchers have determined a positive relationship between overall justice and LMX (Brown et al., 2005). However, few studies have examined the dimensional role of interactional justice in ascertaining the quality of a manager-employee relationship. Interactional justice describes the communication side of organizational justice. As most of the communication between management and employees happens through their immediate bosses, it is argued that the leader’s fair treatment and communication will ultimately strengthen the manager-employee relationship (Piccolo et al., 2008). If a manager is fair to his employees, a strong social exchange relationship is developed between them. It supports the fact that interactional justice has a connection with LMX (Wayne et al., 2002). Therefore, it is argued that if interactional justice exists, there are chances for the development of high-level LMX relationships. Hence it is suggested that:

H_3b_: Interactional Justice positively affects Leader-Member Exchange positively.

### Perceived Organizational Support and Leader-Member Exchange

POS and LMX are the two leading indicators of social exchange in an organization. POS is organization-oriented whereas, LMX is the leader-oriented approach. In some early studies, researchers suggested that organizational support is seen as help from immediate leaders (Shore et al., 1994). Liden et al. (1997) argued that organizational support augments the supervisor-subordinate relationship. Masterson et al. (2000) suggested that the perception of good organizational support promotes a social exchange relationship between supervisors and subordinates. Later on, it was also corroborated by Kurtessis et al. (2017). Based on the evidence, it is asserted that if an employee perceives that his organization supports him, he would likely to build trust in management that will promote and strengthen the social exchange relationship. Support from the organization will be seen as support from the senior management. Therefore, we put forward:

H_4_: Perceived Organizational Support affects Leader-Member Exchange positively.

### Perceived Organizational Support and Readiness for Change

When employees of an organization feel that their organization treats them fairly, and supports them well, they develop a positive perception (Eisenberger et al., 1986). Therefore, employees with a positive perception of organizational support are more likely to welcome any job-related task assigned to them by their employer. In other words, it is here argued that they will tend to develop a sense of readiness for a change. As the change process involves day to day enforcement of actions, organizational support plays a vital role in imparting change readiness (Gigliotti et al., 2019). According to Eisenberger and Stinglhamber (2011), the employees will be more loyal to their organization who feel that they have been supported well by their organization, and are more committed to achieving their organizational goals (Shore and Shore, 1995). POS imparts a sense of responsibility in employees for the organization (Kurtessis et al., 2017). It translates into developing a positive attitude and behavior that might be considered necessary for RFC. Positive perception of organizational support encourages the employees to prepare for the change implementation process (Eby et al., 2000; Mitchell et al., 2012). Thus, it is propounded here that:

H_5a_: Perceived Organizational Support positively affects Readiness for Change.

### Perceived Organizational Support and Resistance to Change

An organization’s support for its employees enhances their commitment level (Rhoades and Eisenberger, 2002). Employees offer their services and, in return, expect incentives, rewards, and social benefits (Armeli et al., 1998). If they are provided with the same, their commitment and loyalty level rise. Employees with more POS are more likely to give their best for the organization, and they will find themselves ready for change initiatives. Therefore, it is asserted that if employees are supported well by their organization, they will be less likely to resist the change process. The Employees of any organization willingly participate in the change process when they conceive that change will prove valuable (Shapiro and Kirkman, 1999). Therefore, drawing from the literature, it is argued that the employees are more likely to lower the resistance toward change when they perceive a strong organizational support (Cummings and Worley, 2009). Hence, we advocate that:

H_5b_: Perceived Organizational Support is negatively related to Resistance for Change.

### Leader-Member Exchange and Readiness for Change

During the change implementation process, the management and employees have to interact daily. So, the quality of subordinate-employee relationships matters a lot in carving the employee’s attitude toward change. Therefore, it can be said that the quality of LMX determines the employee’s intention toward a change initiative. A high-quality LMX relationship among the employees of any organization imparts a sense of loyalty, liking and respect for the leaders because employees in such a relationship are frequently admired for work by their leaders (Brower et al., 2000). The potential rewards of positive behavior development, commitment and trust are associated with a high-level LMX relationship (Karriker and Williams, 2009). Thus, it is argued that LMX supports employee’s RFC. High-quality LMX suggests that the support and trust from the management positively influence employees’ behavioral reactions. Therefore, there are chances that the employees in high-quality LMX relationships develop a positive attitude toward accepting the change (Eby et al., 2000). Therefore, it is inferred that the LMX is strongly related to employee RFC. Consequently, we suggest that:

H_6a_: Leader-member exchange positively affects readiness for change.

### Leader-Member Exchange and Resistance to Change

While reviewing the literature regarding the social exchange and resistance to change, it can be observed that there is an inverse relationship between high-quality LMX and resistance to change. The employees with a high level of LMX relationship are more optimistic toward change-related outcomes. Therefore, they tend to participate in change-related activities instead of posing a resistance (Lee et al., 2010). Biao and Cheng (2014) have highlighted the importance of the leader-employee relationship in the context of resistance to change. If management practices are directed purposefully to make leader-member relationships better, it will significantly help the organization cope with the resistance during the change process (Georgalis et al., 2015). Therefore, it is argued that mutually beneficial supervisor-subordinate relationships facilitate coping with the resistance to change. Hence, it is hypothesized that:

H_6b_: Leader-Member Exchange affects Resistance to Change negatively.

### Readiness for Change and Resistance to Change

If employees of an organization exhibit positive attitudes, beliefs, actions and intentions toward implementing the change, they reflect RFC (French et al., 2005). RFC is very facilitating as it holds primary importance in implementing change. The more the employees are ready for an organizational change, the more they believe in positive change outcomes, consequently increasing the chances of success (Rafferty et al., 2013). Simply, if RFC exists, employees are more likely to accept change rather than resisting it. Therefore, it can be said that their level of resistance to change is reduced to the minimum (Armenakis et al., 1993). So, it turns out that the RFC is an effective predictor of lowering the resistance to change. Therefore, it can be hypothesized that:

H_7_: Readiness for change negatively affects the Resistance to change.

### Mediating Roles of Perceived Organizational Support and Leader-Member Exchange

It has been suggested in the literature that the quality of the relationship between management and employees is essential in dealing with the resistance to change (Ford et al., 2008). Therefore, organizations must concentrate on the factors predicting such high-quality relationships. The social exchange theory (Blau, 1964) explains the organization-employee relationships, and emphasizes how these relationships can be strengthened. In the organizational context, social exchange is a concept of a mutually beneficial relationship between two parties. Therefore, it was advocated that this theory resides on reciprocity norms (Aselage and Eisenberger, 2003). Based on this theory, it was suggested that employee’s perception about the organizational support (POS) establishes a mutual relationship between employee and his organization (Mowday et al., 1982; Mathieu and Zajac, 1990; Meyer and Allen, 1997; Rhoades and Eisenberger, 2002). The researchers have highlighted the importance of POS as it enhances the employee commitment, strengthen his bond with the organization and provide value, in return of the support they receive from their organization (Mowday et al., 1982; Meyer and Allen, 1997; Eisenberger et al., 2001). Here, it is asserted that the employees with POS are anticipated more ready for change and, accordingly, will shape their responses in favor of change instead of resisting it.

Furthermore, it is added that during the change implementation process or otherwise, the employee and his supervisor or boss interact regularly. Therefore, the quality of their mutual relationship matters in achieving the desired organizational outcomes. As discussed earlier, LMX is a phenomenon that refers to the exchange relationship between a supervisor and subordinate (Niehoff et al., 2001). If an employee finds his immediate boss, supervisor, or manager as supportive, he will enthusiastically perform the assigned tasks. It is maintained that a high-quality LMX relationship enables the employee to embrace the organizational change, thereby reducing their resistive attitude toward change. Therefore, organizations need to focus on the factors influencing the quality of LMX. In the context of this study, the literature suggests that employees see their immediate supervisors’ support through the lens of their organization’s support (Shore et al., 1994). Suppose the employees perceive that the organization supports them and realize their efforts. In that case, they will likely build strong exchange relationships with their bosses because they interact daily, and any communication regarding rewards, incentives, on-job training and career counseling mostly happens through immediate bosses. So, employees mostly see the manager’s support as their organization’s support (Kurtessis et al., 2017). Here it can be argued that POS augments LMX, which further makes the employees ready for change, ultimately reducing the resistance to change.

One of the legit reasons behind the failure of a change strategy is the fear of employees about the uncertainty of the future events associated with change. Employees’ confidence, emotions, and behaviors need to be shaped to make them ready for change. Soenen and Melkonian (2017) suggested that justice perceptions greatly influence employee responses in the context of change. Breaking it down to the dimensional level, the perceptions about outcome and rewards distributions (Distributive Justice), procedures adopted for such distributions (Procedural Justice), and how well these distributions and procedures are communicated throughout the organizations (Interactional Justice) influence employee attitude toward change. In this study, it has been formulated that the justice-change relationship is indirect, and there are underlying mechanisms that are mediated by POS and LMX. Moreover, distributive, procedural and interactional justice influence employee’s perception of organizational support (POS) (DeConinck, 2010; Fu and Lihua, 2012). Hence, based on these arguments, it is stated that if an employee perceives justice in the distribution of outcomes, procedures, and management interactions, he will develop positive perceptions about the organization’s support, which will strengthen leader-member relationships. They would build confidence about change, and, finally, resistance to change would reduce considerably. Similarly, distributive, procedural, and interactional justice influence the quality of leader-member relationships (Brown et al., 2005; Lee et al., 2010). Positive the perception about justice dimensions, higher the quality of LMX. Thus, it can be argued that justice dimensions positively impact LMX, which further leads to lowering the resistance to change through employees’ improved state of RFC. So, we propose:

H_8_ (a,b,c,d,e,f): Perceived organizational support mediates the relationships between the dimensions of organizational justice and readiness for change and resistance to change.

H_9_ (a,b,c,d,e,f): Leader-member exchange mediates the relationships between the dimensions of organizational justice and readiness for change and resistance to change.

H_1__0_: Leader-member exchange and readiness for change sequentially mediate the relationship between perceived organizational support and resistance to change.

H_11_ (a,b,c): Perceived organizational support and Leader-Member Exchange sequentially mediate the relationships between the dimensions of organizational justice and readiness for change and resistance to change.

**FIGURE 1:**
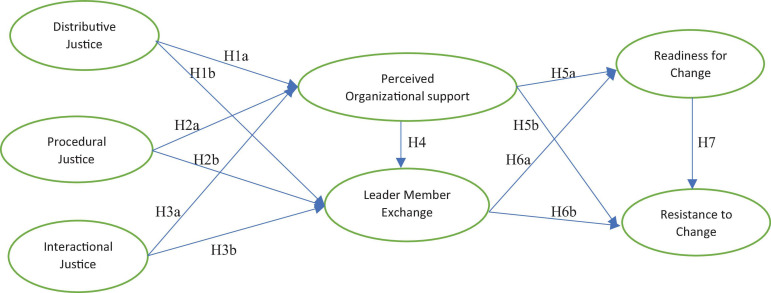
The theoretical model.

Grounded on the proposed hypotheses, we suggest a theoretical research framework chalked out as Figure 1.

## Methodology

### Sample and Procedure

We selected the five largest private commercial banks (based on the number of branches) with a total of 60,311 employees across Pakistan. Furthermore, in these banks, 37,996 employees worked in 3,793 branches across Punjab (the most populated province of Pakistan). The Punjab province was chosen because the majority of bank branches are located in this province. We developed a list of all branches and their employees by obtaining information from the State Bank of Pakistan, and related head offices of banks. The sample was adequately representative of the private banking sector because the top five commercial banks represent all banks in this region, and the data were collected employing a random sampling technique to ensure representativeness. The primary informants for this research were lower and middle managers because they are the frontline workers of banks who deal with implementing and complying with any policy or reform received from top management.

The structured questionnaires were distributed to collect the data. A total of 1,200 questionnaires were distributed across 600 branches, chosen based on a random sampling technique. Out of 1,200 distributed questionnaires, we received 410 questionnaires achieving a response rate of 34.16%. Thirty-eight questionnaires were discarded due to incomplete information, and, hence, the remaining 372 responses were considered for further analysis. The results show that the mean values (μ) of all variables are higher than the corresponding standard deviations (σ). The low values of μ/σ (CV = coefficient of variation) implies that all the variables in our study are under dispersed.

Due to the cross-sectional nature of the study, the findings might be likely to suffer from common method bias due to common method variance (CMV) (Harman, 1976). It is one cause of the correlational error, which arises “when individual responses vary consistently to different degrees over and above true differences in the construct being measured; that is, it is a result of different individuals responding in consistently different ways over and above true differences in the construct” (Viswanathan, 2005, p. 108). This is opposed to random errors of measurement, which are presumed to be independent across the measures of the same or different constructs (Baumgartner et al., 2021). Both ex-ante and ex-post approaches were used to restrict CMV. The following remedies were adopted during the research design stage as an ex-ante approach: (a): Assuring the secrecy and anonymity, the respondents were stressed upon providing fair responses disregarding them right or wrong (Podsakoff et al., 2003); (b): the items of all constructs (independent, dependent and mediators) were shuffled to prevent a biased pattern of ticking the anchors in “creating” the correlation (Murray et al., 2005); (c): the construction of the complex model in anticipation to avoid the mental model of interactions (Harrison et al., 1996).

After that, statistical analyses were conducted to assess CMV as an ex-post approach. First of all, the most reported post hoc test, Harman’s single factor, was conducted without rotating the factor. The test resulted in a 29% variance explained by the single factor, which is less than the prevailing threshold value of 50%. It means no single factor emerged, and hence, there was no existence of CMV in the data. However, Podsakoff et al. (2003, 2012) explain that the test has a low sensitivity in detecting CMV because it is implausible that a single-factor model would fit the complete data (notably, in the absence of some useful threshold). Due to the shortcoming of Harman’s single factor test, Podsakoff et al. (2012) recommend testing the measurement model (CFA) with and without a single latent factor, called a common latent factor (CLF). A CLF is a latent factor showing direct links with all the indicators (items). Hence, CFA was run with and without CLF, and both the measurement models achieved good fits. Now, in order to detect CMV, the standardized loadings of the two models were compared. The difference between these loadings was found to be less than 0.2, implying that CMV did not significantly inflate the estimates of the model CLF was not specified (Devonish, 2018). Thus, the presence of CMV was disregard in this study.

The attributes of the study sample have been described in Table 1. It reveals that the majority of the respondents were male. The dominant group of respondents was lower management with ages between 31 and 40 years and experience of more than 10 years.

**TABLE 1:** Demographic characteristics of the respondents.

Attribute	Frequency	Percent	Cumulative percent
**Gender**			
Male	341	91.67	91.67
Female	31	8.33	100
**Age**			
21–30 years	43	11.56	11.56
31–40 years	219	58.87	70.47
41–50 years	86	23.11	93.58
Above 50 years	24	6.46	100
**Experience**			
Less than or equal to 10 years	259	69.62	69.62
11–20 years	67	18.01	87.63
Above 20 years	46	12.37	100
**Designation**			
Lower management	323	86.83	86.83
Middle management	49	13.17	100

### Measures

A seven-point Likert scale with a range from 1 = strongly disagree to 7 = strongly agree was used. The respondents were asked to rate the degree to which they agree or disagree with a particular statement. The organizational justice scale developed by Niehoff and Moorman (1993) was used, which contains five items for distributive justice, six items for procedural justice, and nine items for interactional justice. A seven-item scale developed by Scandura and Graen (1984), also known as LMX-7, was used to measure LMX. An eight-item scale developed by Rhoades and Eisenberger (2002) was used to collect POS data. Furthermore, a nine-item scale by Bouckenooghe et al. (2009) was used for RFC. Similarly, an eighteen-item scale developed by Oreg (2003) was used to collect responses for RTC. All the items used in the study have been placed in Supplementary Appendix for reference.

### The Measurement Model (Confirmatory Factor Analysis)

A partial least squares structural equation modeling (PLS-SEM) technique has been employed through SmartPLS 3.2.6 to analyze the research framework due to the non-normality of the sample collected. PLS-SEM proposes maximizing the dependent variables’ predictive accuracy while allowing the constructs to retain more items. This method was preferred because (1) data normal distribution is not necessary; (2) PLS-SEM is acceptable for the predictive purpose; and (3) it deals with the complexity of the model in terms of hypothesized relations and variables (Albort-Morant and Ribeiro-Soriano, 2016).

The internal consistency of the constructs was measured by the values of Cronbach’s alpha and composite reliability (CR), as shown in Table 2. The reliability was established because the values were above the acceptable threshold of 0.7 (Gefen et al., 2000).

**TABLE 2:** The measurement model.

Constructs	Cronbach’s alpha	CR	AVE	Loading	VIF range
DJ	0.905	0.929	0.725	0.903–0.805	2.11–3.47
PJ	0.876	0.907	0.619	0.866–0.729	1.79–2.96
IJ	0.928	0.940	0.636	0.872–0.706	2.39–4.37
POS	0.927	0.940	0.663	0.860–0.772	3.05–4.42
LMX	0.936	0.949	0.725	0.915–0.792	2.32–4.86
RFC	0.931	0.942	0.644	0.841–0.756	1.94–4.93
RTC	0.969	0.972	0.658	0.901–0.694	2.13–4.21

*DJ, Distributive Justice; PJ, Procedural Justice; IJ, Interactional Justice; POS, Perceived Organizational Support; LMX, Leader Member Exchange; RFC, Readiness for Change; RTC, Resistance to Change.*

**FIGURE 2:**
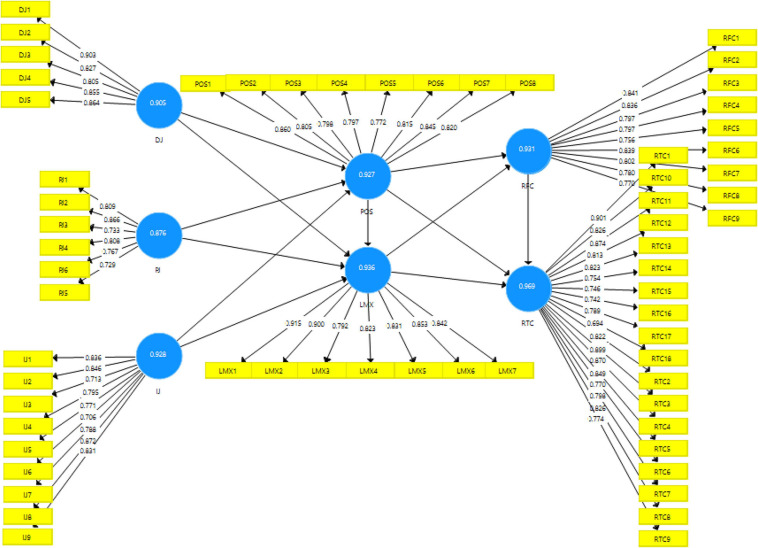
Factor loadings.

Similarly, convergent validity and discriminant validity are the two ways that assess construct validity (O’Leary-Kelly and Vokurka, 1998). The convergent validity was analyzed by investigating factor loadings and Average Variance Extracted (AVE) values of the measures. AVE, explained by a latent construct, shows the complete variance of indicators (Fornell and Larcker, 1981). The values of loadings and AVE were above the threshold levels of 0.7 and 0.5, respectively (Gliem and Gliem, 2003), as shown in Figure 2 and Table 2.

Then discriminant validity, which assesses the degree of variation of one construct from others, was measured by ascertaining that the square roots of AVE values must be higher than the correlation between constructs (Fornell and Larcker, 1981). Table 3 demonstrates that square roots of AVE values of constructs (shown in the diagonal) are greater than the inter-construct correlations (off-diagonal elements), establishing the discriminant validity. The significant values of correlations have also been reported here. Further, Table 3 displays the mean and standard deviation values, which show a narrow spread of the data.

**TABLE 3:** Discriminant validity.

	Mean	SD	DJ	IJ	LMX	PJ	POS	RFC	RTC
DJ	5.69	0.94	0.852						
IJ	5.65	0.79	0.399**	0.797					
LMX	5.78	0.89	0.495**	0.420*	0.852				
PJ	5.54	0.93	0.596**	0.429**	0.505**	0.787			
POS	5.26	0.78	0.496**	0.412**	0.592**	0.530**	0.814		
RFC	5.71	0.80	0.514**	0.422**	0.567**	0.633**	0.593**	0.803	
RTC	2.43	1.01	−0.348*	−0.250*	−0.478**	−0.398*	−0.440**	−0.474**	0.811

*p < 0.05, **p < 0.01.

*The square roots of AVE (Average Variance Extracted) of constructs are shown in the diagonal, while inter-construct correlations are shown as off-diagonal elements.*

### Structural Model

**FIGURE 3:**
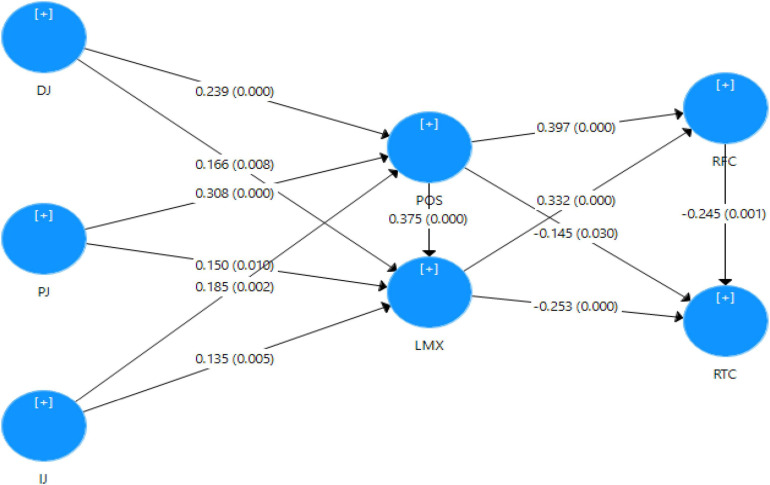
Structural model.

After acceptable and appropriate results of the measurement model, the study analyzed the research hypotheses through the PLS-SEM approach. The empirical results of the structural model have been presented in Figure 3.

The structural model presents direct relationships related to12 research hypotheses, as reported in Table 4. POS is significantly influenced by DJ (β = 0.239, *p* < 0.000), PJ (β = 0.308, *p* < 0.000), and IJ (β = 0.185, *p* < 0.001), which support the hypotheses H_1a_, H_2a_, and H_3__a_, respectively. Hence, the results show that the dimensions of organizational justice have a significant positive effect on POS. However, IJ has the least positive significant relationship with POS. Similarly, LMX is significantly influenced by DJ (β = 0.166, *p* < 0.006), PJ (β = 0.150, *p* < 0.008), and IJ (β = 0.135, *p* < 0.007), which support hypotheses H_2a_, H_2b_, and H_3__c_, respectively. The results demote that the dimensions of organizational justice have a significant positive influence on LMX. The results for H_4_ show that POS positively affects LMX (β = 0.375, *p* < 0.000). In addition, H_5a_, H_6a_, H_5b_, and H_6b_ have also been supported, which show that RFC is positively influenced by POS (β = 0.397, *p* < 0.000) and LMX (β = 0.332, *p* < 0.000). Furthermore, RTC is significantly and negatively influenced by POS (β = −0.145, *p* < 0.032) and LMX (β = −0.253, *p* < 0.000). In the end, H_7_ has also been supported (β = 0.245, *p* < 0.001), representing that RFC has a significant negative impact on RTC.

**TABLE 4:** Direct path coefficient.

Hypotheses	Relationship	Path coefficient	T-statistics	*P*-values	Direction
H1a	DJ - > POS	0.239	3.951	0.000	Supported
H1b	DJ - > LMX	0.166	2.750	0.006	Supported
H2a	PJ - > POS	0.308	5.565	0.000	Supported
H2b	PJ - > LMX	0.150	2.679	0.008	Supported
H3a	IJ - > POS	0.185	3.300	0.001	Supported
H3b	IJ - > LMX	0.135	2.689	0.007	Supported
H4	POS - > LMX	0.375	4.335	0.000	Supported
H5a	POS - > RFC	0.397	8.147	0.000	Supported
H5b	POS - > RTC	−0.145	2.150	0.032	Supported
H6a	LMX - > RFC	0.332	7.250	0.000	Supported
H6b	LMX - > RTC	−0.253	4.209	0.000	Supported
H7	RFC - > RTC	−0.245	3.430	0.001	Supported

Similarly, this study has utilized the Hayes (2018) process to analyze mediation because it does not strictly assume distribution (Hair et al., 2013). Hayes (2018) process utilizes the bootstrapping technique in two steps. First of all, the significance level of a direct relationship is checked by employing bootstrapping, in which the mediating variable is not present in the model. Afterward, the significance of indirect effect and associated *t*-values are checked when the mediator is included in the model. The results of the mediation analysis have been presented in Table 5.

**TABLE 5:** The indirect effects.

Hypotheses	Relationship	Path coefficient	T statistics	*P*-values	Decision
H8a	DJ - > POS - > LMX - > RFC	0.030	3.035	0.003	Supported
H8b	PJ - > POS - > LMX - > RFC	0.038	3.155	0.002	Supported
H8c	IJ - > POS - > LMX - > RFC	0.023	2.316	0.021	Supported
H8d	DJ - > POS - > LMX - > RTC	−0.023	2.464	0.014	Supported
H8e	PJ - > POS - > LMX - > RTC	−0.029	2.585	0.010	Supported
H8f	IJ - > POS - > LMX - > RTC	−0.018	2.054	0.040	Supported
H9a	DJ - > LMX - > RFC - > RTC	−0.013	1.859	0.064	Not Supported
H9b	PJ - > LMX - > RFC - > RTC	−0.012	1.859	0.064	Not Supported
H9c	IJ - > LMX - > RFC - > RTC	−0.011	1.949	0.052	Supported
H9d	DJ - > POS - > RFC - > RTC	−0.023	2.272	0.024	Supported
H9e	PJ - > POS - > RFC - > RTC	−0.030	2.581	0.010	Supported
H9f	IJ - > POS - > RFC - > RTC	−0.018	2.026	0.043	Supported
H10	POS - > LMX - > RFC - > RTC	−0.030	2.328	0.020	Supported
H11a	DJ - > POS - > LMX - > RFC - > RTC	−0.007	2.045	0.041	Supported
H11b	PJ - > POS - > LMX - > RFC - > RTC	−0.009	2.145	0.032	Supported
H11c	IJ - > POS - > LMX - > RFC - > RTC	−0.006	1.789	0.074	Not Supported

The analysis results show that intervening variables mediate most of the relationships between organizational justice dimensions and resistance to change, as explained subsequently. This study analyzed the sequential role of POS and LMX between justice dimensions (DJ, PJ, and IJ) and RFC. The results show that the paths from justice dimensions (DJ, PJ, and IJ) to POS → LMX → RFC are significant. Each dimension of organizational justice reaches to RFC significantly through POS and LMX (β = 0.030, *t* = 3.035, *p* = 0.003), (β = 0.038, *t* = 3.155, *p* = 0.002), and (β = 0.023, *t* = 2.316, *p* = 0.021). This represents that hypotheses: H_8a_, H_8b_, and H_8c_ are supported. Then the study examined the sequential role of POS and LMX between justice dimensions (DJ, PJ, and IJ) and RTC. The results show that each dimension of organizational justice reaches to RTC significantly through POS and LMX (β = −0.023, *t* = 2.464, *p* = 0.014), (β = −0.029, *t* = 2.585, *p* = 0.010), and (β = −0.018, *t* = 2.054, *p* = 0.040). This represents that hypotheses: H_8d_, H_8e_, and H_8f_ of our study are supported. Furthermore, the paths from justice dimensions DJ, PJ, and IJ to RTC through LMX and RFC (DJ, PJ, and IJ) to LMX → RFC → RTC have been analyzed. The results indicated that the paths from DJ, PJ do not reach significantly to RTC (β = −0.013, *t* = 1.859, *p* = 0.064), (β = −0.012, *t* = 1.859, *p* = 0.064), whereas the path: IJ → LMX → RFC → RTC is significant but with very low significance level (β = −0.011, *t* = 1.949, *p* = 0.052). This suggests that hypotheses H_9a_ and H_9b_ are not supported, whereas H_9c_ finds moderate support. Furthermore, hypotheses for relationships (DJ, PJ, IJ) to POS → RFC → RTC for hypotheses H_9d_, H_9e_, and H_9f_ are supported. H_10_ has also been tested as significant (β = −0.030, *t* = 2.328, *p* = 0.020). Finally, the study has analyzed the final three paths that reach from DJ, PJ, and IJ to RTC with the sequential role of POS, LMX and RFC. The paths are from justice dimensions (DJ, PJ, and IJ) to POS → LMX → RFC → RTC. The results have indicated that POS, LMX and RFC sequentially mediate the relationship of DJ, PJ with RTC (β = −0.007, *t* = 2.045, *p* = 0.041), (β = −0.009, *t* = 2.145, *p* = 0.032), supporting the hypotheses H_1__1a_ and H_1__1b_. For IJ—the third dimension of organizational justice, the path does not reach RTC significantly through POS, LMX, and RFC (β = s−0.006, *t* = 1.789, *p* = 0.074), representing that the last hypothesis of our study, H_1__1c_, is not supported.

## Discussion and Conclusion

This study aims to extend the literature by demonstrating how different paths can lower the resistance to change in organizational justice and social exchange relationships within an organization. The study contributes to the literature by considering data from various branches of multiple banks from Pakistan. It has provided us with a broader view and clear assessment of antecedents and their effects on dependent constructs of the study (Oreg et al., 2011). In previous organizational change-related studies, data were collected mainly from a single organization. Furthermore, there is a lack of research where the data were gathered from different organizations or organizations with multiple branches (Fedor et al., 2006; Banguntopo, 2018). This study mitigates the shortcomings and contributes to the theory by investigating the dimensions of organizational justice and their role in determining the most suitable path in thwarting the employee resistance to change through POS, LMX, and RFC.

The results of our study have presented that all of the direct hypotheses are supported. It has been established that the level of organizational justice being practiced by the organization determines the quality of social exchange relationships (Bauer and Green, 1996) between employees and management as well as between employees and their organization. Organizational justice not only impacts employee productivity but also influences several important organizational outcomes (Fiaz et al., 2021). The results of the current study have supported that the dimensions of organizational justice (distributive justice, procedural justice, and interactional justice) positively impact the quality of POS and LMX. The fair process of distribution of rewards and adoption of equitable procedures enhances POS and LMX. These findings are aligned with previous studies (DeConinck, 2010; Lee et al., 2010; Fu and Lihua, 2012).

Similarly, the results have shown that interactional justice strongly affects POS and LMX. An organization’s fair communications and interactions with the employees positively influence superior-subordinate relationships (Piccolo et al., 2008). Then, the result of hypothesis H_4_ represents that POS positively influences LMX. As suggested by the previous literature, employees perceive their organization’s support as their manager’s support, and, as a result, they form high-quality social exchange relationships with their boss (Shore et al., 1994). The social exchange relationships (POS and LMX) induce positive perceptions regarding change outcomes, resulting in RFC among the employees. The findings are convergent with the literature (Eby et al., 2000). Moreover, the study has observed that the more the employees are ready for change, the less chance they show negative responses, and less will be the change-resistant. This is also in line with the established literature on change (Armenakis et al., 1993; Rafferty et al., 2013).

Likewise, the findings have also demonstrated a significant impact of dimensions of organizational justice (distributive justice, procedural justice, and interactional justice) on resistance to change (RTC) through mediators. Furthermore, the results represent that this effect is mediated sequentially through POS, LMX, and RFC. When dimensions of organizational justice are discussed individually, they significantly affect RTC when moving through the path from POS, LMX, and RFC. One path has been resulted completely insignificant, that is, IJ - > POS - > LMX - > RFC - > RTC. Furthermore, three other paths were found relatively less significant: **1.** DJ - > LMX - > RFC - > RTC, **2.** IJ - > LMX - > RFC - > RTC, and **3.** PJ - > LMX - > RFC - > RTC due to longer paths. All the other paths are significant, ranging from moderate to very good level of significance, as displayed by p values representing the strength of indirect relationships (as represented in Table 4). This finding provides us with important insights regarding the positioning of justice dimensions as antecedents of RTC in an indirect framework through POS, LMX and RFC.

Since most employees fear that change will not bring any good for them or the organization itself (Arnéguy et al., 2018), managers are concerned about lowering such a resistive attitude. Hence, the introduction of a comprehensive, sequential framework is a dire need of the current times. As a conclusion of this research, it has been put forward that employee resistance can be dealt with by making them ready for change. Acceptance for change becomes more promising when employees receive considerable support from their organization (POS) as well as from their managers (LMX). When the earlier discussed relationships are present, they will positively impact the state of an employee’s readiness for an organizational change, which will then be translated into “minimization of change resistance.” That will significantly help in the successful implementation of change.

### Practical Implications

Change management has become one of the most important business priorities because organizations need to stay up-to-date to compete in today’s business environment. Organizations need to understand how to make their employees ready for the change to successfully implement a change strategy (Ferlie et al., 2005). This study has provided us with a bird’s-eye view about the dimensional role of organizational justice in minimizing the resistance to change, indirectly through POS, LMX, and RFC. Suppose an organization intends to devise a change management strategy, as suggested by the empirical evidence of this study. In that case, it needs to focus on how and their distribution and procedures bring can justice among the employees because it is a building block of employee’s perception of organizational support and superior-subordinate social exchange relations (Colquitt et al., 2013; Georgalis et al., 2015). Thus, employees will participate in change-oriented assignments willingly if they realize that change is beneficial for them. It is suggested that such a sense of RFC is better derived by organizational justice. Organizations may incorporate the underlying mechanisms discussed in this study as a tool for policymaking. For instance, an organization’s focus on distributive justice and procedural justice will catalyze organizational support, which will lead to a high-level LMX relationship. Subsequently, it will lead to minimizing the resistance to change through RFC. Undermining the role of organizational support, subordinate-employee relationships, or a sense of readiness for unimagined change may cost the organization a lot. If employee resistance to change is compromised, an organization may fail in its change implementation effort (Meaney and Pung, 2008), leaving it with substantial financial losses.

The research framework tested and discussed in this study would prove to be a valuable tool for the management in understanding and designing a system to help them deal with the resistance to change. The success of any organization mainly depends on human capital because the employees are often treated as assets by successful organizations. In this regard, the studies relating to the employee’s behaviors and attitudes hold distinctive importance. Therefore, in terms of organizational change, the POS and quality of leader-member relationships play a vital role in determining the decisive approach of employees.

### Limitations and Future Recommendations

Like other studies, our study is prone to several limitations that point to future directions for the researchers. First, our research has adopted a cross-sectional framework; however, a longitudinal approach for data collection will significantly contribute to the literature of change and organizational justice by providing insights into cause-and-effect relationships. Since this study is related to human resources and organizational behavior, data collected over time will significantly contribute significantly to the change management literature. Moreover, data were collected from the banking industry only. Change management is a big concern for other sectors as well, such as the manufacturing industry. Therefore, it will be valuable to use the small and large manufacturing industries as the sampling frame. Furthermore, since the current study extends recent change literature by exploring the justice dimensions, it is suggested that the effect of these dimensions on resistance to change may further be studied through other indirect variables like organizational identification, organization citizenship behavior. In this way, the researchers may determine a more favorable and robust framework with reference to antecedents and outcomes of change management.

## Data Availability Statement

The raw data supporting the conclusions of this article will be made available by the authors, without undue reservation.

## Ethics Statement

Ethical review and approval was not required for the study on human participants in accordance with the local legislation and institutional requirements. The patients/participants provided their written informed consent to participate in this study.

## Author Contributions

NR, AM, and SAM contributed to the conception and design of the study. NI organized the database. MI and EM performed the statistical analysis. NR wrote the first draft of the manuscript. AM, EM, MI, NI, and SAM wrote sections of the manuscript. All authors contributed to manuscript revision, read, and approved the submitted version.

## Conflict of Interest

The authors declare that the research was conducted in the absence of any commercial or financial relationships that could be construed as a potential conflict of interest.

## Publisher’s Note

All claims expressed in this article are solely those of the authors and do not necessarily represent those of their affiliated organizations, or those of the publisher, the editors and the reviewers. Any product that may be evaluated in this article, or claim that may be made by its manufacturer, is not guaranteed or endorsed by the publisher.
