# Supplementary material for: The Psychology of Resistance to Change: The Antidotal Effect of Organizational Justice, Support and Leader-Member Exchange
Source: Front Psychol. 2021 Aug 2;12:678952. doi: 10.3389/fpsyg.2021.678952 (PMC8365138; doi:10.3389/fpsyg.2021.678952)
Supplement: Supplementary file 1 [file Data_Sheet_1.docx]

# **Appendix**

### **The Instrument**

1. **Organizational Justice**

1= Strongly Disagree, 2= Disagree, 3= Slightly Disagree, 4= Neutral, 5= Slightly Agree, 6= Agree, 7= Strongly Agree

| **Distributive Justice** | | | | | | | |
| --- | --- | --- | --- | --- | --- | --- | --- |
| My work schedule is fair. | 1 | 2 | 3 | 4 | 5 | 6 | 7 |
| I think that my level of pay is fair. | 1 | 2 | 3 | 4 | 5 | 6 | 7 |
| I consider my work load to be quite fair | 1 | 2 | 3 | 4 | 5 | 6 | 7 |
| Overall, the rewards I receive here are quite fair. | 1 | 2 | 3 | 4 | 5 | 6 | 7 |
| I feel that my job responsibilities are fair. | 1 | 2 | 3 | 4 | 5 | 6 | 7 |

| **Procedural Justice** | | | | | | | |
| --- | --- | --- | --- | --- | --- | --- | --- |
| Job decisions are made by the management in an unbiased manner. | 1 | 2 | 3 | 4 | 5 | 6 | 7 |
| The management makes sure that all employee concerns are heard before job decisions are made. | 1 | 2 | 3 | 4 | 5 | 6 | 7 |
| To make job decisions, the management collects accurate and complete information. | 1 | 2 | 3 | 4 | 5 | 6 | 7 |
| The management clarifies decisions and provides additional information when requested by employees. | 1 | 2 | 3 | 4 | 5 | 6 | 7 |
| All job decisions are applied consistently across all affected employees. | 1 | 2 | 3 | 4 | 5 | 6 | 7 |
| Employees are allowed to challenge or appeal job decisions made by the management. | 1 | 2 | 3 | 4 | 5 | 6 | 7 |

| **Interactional Justice** | | | | | | | |
| --- | --- | --- | --- | --- | --- | --- | --- |
| When decisions are made about my job, the management treats me with kindness and consideration. | 1 | 2 | 3 | 4 | 5 | 6 | 7 |
| When decisions are made about my job, the management treats me with respect and dignity. | 1 | 2 | 3 | 4 | 5 | 6 | 7 |
| When decisions are made about my job, the management is sensitive to my personal needs. | 1 | 2 | 3 | 4 | 5 | 6 | 7 |
| When decisions are made about my job, the management deals with me in a truthful manner. | 1 | 2 | 3 | 4 | 5 | 6 | 7 |
| When decisions are made about my job, the management shows concern for my rights as an employee. | 1 | 2 | 3 | 4 | 5 | 6 | 7 |
| Concerning decisions made about my job. the management discusses the implications of the decisions with me. | 1 | 2 | 3 | 4 | 5 | 6 | 7 |
| The management offers adequate justification for decisions made about my job. | 1 | 2 | 3 | 4 | 5 | 6 | 7 |
| When making decisions about my job. the management offers explanations that make sense to me. | 1 | 2 | 3 | 4 | 5 | 6 | 7 |
| The management explains very clearly any decision made about my job. | 1 | 2 | 3 | 4 | 5 | 6 | 7 |

1. **Perceived Organizational Support**

1= Strongly Disagree, 2= Disagree, 3= Slightly Disagree, 4= Neutral, 5= Slightly Agree, 6= Agree, 7= Strongly Agree

| The organization values my contribution to its well-being. | 1 | 2 | 3 | 4 | 5 | 6 | 7 |
| --- | --- | --- | --- | --- | --- | --- | --- |
| The organization fails to appreciate any extra effort from me. | 1 | 2 | 3 | 4 | 5 | 6 | 7 |
| The organization would ignore any complaint from me. | 1 | 2 | 3 | 4 | 5 | 6 | 7 |
| The organization really cares about my well-being. | 1 | 2 | 3 | 4 | 5 | 6 | 7 |
| Even if I did the best job possible, the organization would fail to notice. | 1 | 2 | 3 | 4 | 5 | 6 | 7 |
| The organization cares about my general satisfaction at work. | 1 | 2 | 3 | 4 | 5 | 6 | 7 |
| The organization shows very little concern for me. | 1 | 2 | 3 | 4 | 5 | 6 | 7 |
| The organization takes pride in my accomplishments at work. | 1 | 2 | 3 | 4 | 5 | 6 | 7 |

1. **Leader-Member Exchange**

1= Strongly Disagree, 2= Disagree, 3= Slightly Disagree, 4= Neutral, 5= Slightly Agree, 6= Agree, 7= Strongly Agree

| My working relationship with my supervisor is very effective. | 1 | 2 | 3 | 4 | 5 | 6 | 7 |
| --- | --- | --- | --- | --- | --- | --- | --- |
| I always know how satisfied my supervisor is with what I do. | 1 | 2 | 3 | 4 | 5 | 6 | 7 |
| My supervisor would use his/her power to help me solve work-related problems. | 1 | 2 | 3 | 4 | 5 | 6 | 7 |
| I always know where I stand with my supervisor. | 1 | 2 | 3 | 4 | 5 | 6 | 7 |
| My manager understands my job problems and needs. | 1 | 2 | 3 | 4 | 5 | 6 | 7 |
| My manager recognizes my potential well. | 1 | 2 | 3 | 4 | 5 | 6 | 7 |
| My supervisor would “bail me out” at his/her expense. | 1 | 2 | 3 | 4 | 5 | 6 | 7 |

1. **Readiness for Change**

1= Strongly Disagree, 2= Disagree, 3= Slightly Disagree, 4= Neutral, 5= Slightly Agree, 6= Agree, 7= Strongly Agree

| I have a good feeling about the change project | 1 | 2 | 3 | 4 | 5 | 6 | 7 |
| --- | --- | --- | --- | --- | --- | --- | --- |
| I experience the change as a positive process | 1 | 2 | 3 | 4 | 5 | 6 | 7 |
| I find the change refreshing. | 1 | 2 | 3 | 4 | 5 | 6 | 7 |
| Plans for future improvement will not come too much. | 1 | 2 | 3 | 4 | 5 | 6 | 7 |
| I want to devote myself to the process of change. | 1 | 2 | 3 | 4 | 5 | 6 | 7 |
| I think that most changes will have a negative effect on the customers/clients we serve | 1 | 2 | 3 | 4 | 5 | 6 | 7 |
| Most change projects that are supposed to solve problems around here will not do much good. | 1 | 2 | 3 | 4 | 5 | 6 | 7 |
| I am willing to make a significant contribution to the change. | 1 | 2 | 3 | 4 | 5 | 6 | 7 |
| I am willing to put energy into the process of change | 1 | 2 | 3 | 4 | 5 | 6 | 7 |

1. **Resistance for Change**

1= Strongly Disagree, 2= Disagree, 3= Slightly Disagree, 4= Neutral, 5= Slightly Agree, 6= Agree, 7= Strongly Agree

| I generally consider changes to be a negative thing. | 1 | 2 | 3 | 4 | 5 | 6 | 7 |
| --- | --- | --- | --- | --- | --- | --- | --- |
| I’ll take a routine day over a day full of unexpected events any time. | 1 | 2 | 3 | 4 | 5 | 6 | 7 |
| I like to do the same old things rather than try new and different ones. | 1 | 2 | 3 | 4 | 5 | 6 | 7 |
| Whenever my life forms a stable routine, I look for ways to change it. | 1 | 2 | 3 | 4 | 5 | 6 | 7 |
| I’d rather be bored than surprised. | 1 | 2 | 3 | 4 | 5 | 6 | 7 |
| If I were to be informed that there’s going to be a significant change regarding the way things are done at work, I would probably feel stressed. | 1 | 2 | 3 | 4 | 5 | 6 | 7 |
| When I am informed of a change of plans, I tense up a bit. | 1 | 2 | 3 | 4 | 5 | 6 | 7 |
| When things don’t go according to plans, it stresses me out. | 1 | 2 | 3 | 4 | 5 | 6 | 7 |
| If my boss changed the criteria for evaluating employees, it would probably make me feel uncomfortable even if I thought I’d do just as well without having to do any extra work. | 1 | 2 | 3 | 4 | 5 | 6 | 7 |
| Changing plans seems like a real hassle to me. | 1 | 2 | 3 | 4 | 5 | 6 | 7 |
| Often, I feel a bit uncomfortable even about changes that may potentially improve my life. | 1 | 2 | 3 | 4 | 5 | 6 | 7 |
| When someone pressures me to change something, I tend to resist it even if I think the change may ultimately benefit me. | 1 | 2 | 3 | 4 | 5 | 6 | 7 |
| I sometimes find myself avoiding changes that I know will be good for me. | 1 | 2 | 3 | 4 | 5 | 6 | 7 |
| Once I’ve made plans, I’m not likely to change them. | 1 | 2 | 3 | 4 | 5 | 6 | 7 |
| I often change my mind. | 1 | 2 | 3 | 4 | 5 | 6 | 7 |
| Once I’ve come to a conclusion, I’m not likely to change my mind. | 1 | 2 | 3 | 4 | 5 | 6 | 7 |
| I don’t change my mind easily. | 1 | 2 | 3 | 4 | 5 | 6 | 7 |
